# HBV Dominance Is Associated With a Distinct Inflammatory Milieu in HBV/HCV Coinfection

**DOI:** 10.1111/jvh.70092

**Published:** 2025-10-15

**Authors:** Carlos Oltmanns, Moana Witte, Anika Wranke, Katja Deterding, Heiner Wedemeyer, Christine S. Falk, Anke R. M. Kraft, Steffen B. Wiegand, Markus Cornberg

**Affiliations:** ^1^ Department of Gastroenterology, Hepatology, Infectious Diseases and Endocrinology Hannover Medical School (MHH) Hannover Germany; ^2^ Centre for Individualised Infection Medicine (CiiM), a Joint Venture Between the Helmholtz Centre for Infection Research (HZI) and Hannover Medical School (MHH) Hannover Germany; ^3^ TWINCORE, a Joint Venture Between the Helmholtz‐Centre for Infection Research (HZI) and the Hannover Medical School (MHH) Hannover Germany; ^4^ German Centre for Infection Research (DZIF), Partner Site Hannover‐Braunschweig Germany; ^5^ HepNet Study‐House/German Liver Foundation Hannover Germany; ^6^ Cluster of Excellence RESIST (EXC 2155), Hannover Medical School Hannover Germany; ^7^ Institute of Transplant Immunology Hannover Medical School Hannover Germany; ^8^ Department of Anesthesiology and Intensive Care Medicine Hannover Medical School Hannover Germany

**Keywords:** cirrhosis, coinfection, cytokine, fibrosis, HBV activity, HCC, IL‐17, inflammation, JAK–STAT, Th17

## Abstract

Hepatitis B (HBV) and C (HCV) virus coinfection is linked to a higher risk of cirrhosis and hepatocellular carcinoma (HCC) compared to monoinfection. Despite this, data are limited, and further investigation is needed to understand the underlying mechanisms. While patients are classified based on dominance patterns, the impact on the immune system remains largely unknown. It is recognised that HBV reactivation may occur following HCV clearance. This study aims to explore the potential immune interactivity between HCV and HBV by analysing patterns of soluble immune mediators (SIM). A total of 58 soluble immune mediators were measured in serum or plasma samples of 49 patients chronically infected with hepatitis B and hepatitis C virus in a cross‐sectional study design. Patients were classified based on dominance patterns: HBV dominance (*n* = 8), HCV dominance (*n* = 22), HBV and HCV codominance (*n* = 11) and no dominance (*n* = 8). SIM expression was distinct based on different dominance patterns. HBV activity induced higher SIM expression and altered the soluble inflammatory milieu (22 SIM altered, *p* < 0.05). Altered pathways included JAK–STAT pathway (*p* = 1.36 × 10^−20^), IL‐17 signalling (*p* = 2.47 × 10^−13^) and Th17 cell differentiation (*p* = 1.69 × 10^−9^). CCL27/CTACK (*r* = −0.69, *p* = 7.02 × 10^−6^) and SDF‐1alpha (*r* = −0.55, *p* = 0.002) correlated inversely with HCV‐RNA. Serologically classifying dominance patterns in HBV and HCV coinfection may manifest in a distinct soluble inflammatory milieu. Elevated HBV activity correlates with an increased expression of soluble immune mediators, particularly influencing the alteration of key signalling pathways such as JAK–STAT and the Th17/IL‐17 axis. These changes have a potential role in the development of liver fibrosis.

**Abbreviations**
ALT: Alanine amino transferase
AST: Aspartate aminotransferase
DAA: Direct‐acting antiviral
HBsAg: HBV surface antigen
HBV: Hepatitis B virus
HCC: Hepatocellular carcinoma
HCV: Hepatitis C virus
KEGG: Kyoto Encyclopedia of Genes and Genomes
NA: Nucleoside/nucleotide analogue
PCA: Principal component analysis
RT: Room temperature
SIM: Soluble immune mediators

## 1 Introduction

Hepatitis B virus (HBV) and hepatitis C virus (HCV) infections represent significant global health challenges [1, 2]. Individuals with chronic HBV or HCV infections face various liver‐related complications, including cirrhosis and hepatocellular carcinoma (HCC) [3]. Coinfection with HBV and HCV is associated with an even higher risk for the development of cirrhosis and HCC than monoinfection alone [4, 5]. Tyson et al. estimated the prevalence of coinfection among people chronically infected with HCV at around 1.4% in the US [6] with varying rates in Egypt [7] and Taiwan [8] ranging from 0.7% to 12% of HCV patients being chronically infected with HBV. Given the shared transmission routes, rates may be even higher in individuals with intravenous drug use (PWID) and men who have sex with men [9, 10]. The infection rates have to be carefully evaluated since recent studies on this subject are limited in number and the introduction of widespread HBV vaccination will likely change this epidemiology [11].

Since 2014, the widespread use of direct‐acting antiviral (DAA) therapies has revolutionised HCV treatment, achieving more than 98% efficacy [12]. A noteworthy consequence of successful hepatitis C treatment is the reactivation of HBV infection in coinfected patients [13]. Guidelines recommend a nucleoside/nucleotide analogue (NA) prophylaxis for HCV patients coinfected with HBV undergoing anti‐HCV DAA treatment [14]. A possible explanation for HBV reactivation in HCV/HBV co‐infected patients after DAA therapy could be reduced hepatic type I interferon responses, which were initially triggered by HCV and are reduced after cure [15], though the precise mechanisms of this common complication warrant further investigation [13].

Patients coinfected with HBV and HCV can be classified into four different dominance groups based on their serological profile, but some might change their pattern over time [16, 17]. Interestingly, most patients coinfected with HBV and HCV show an HCV dominance pattern with a high HCV‐RNA and low HBV‐DNA [18]. In the majority of coinfections, HCV suppresses HBV replication [13, 15, 19]. While cytokines such as CXCL‐10 are associated with this dominance pattern [20], the exact mechanisms governing the interplay between both viruses remain elusive.

This study aimed to investigate the expression of 58 soluble immune mediators (SIM) in the context of HBV and HCV coinfection. Utilizing a cross‐sectional analysis based on established dominance patterns, we provide new insights into the immunological interactions during HBV and HCV coinfection. Ultimately, our results underscore a previously underestimated role of HBV activity in patients coinfected with HBV and HCV.

## 2 Materials and Methods

### 2.1 Study Population and Design

**FIGURE 1:**
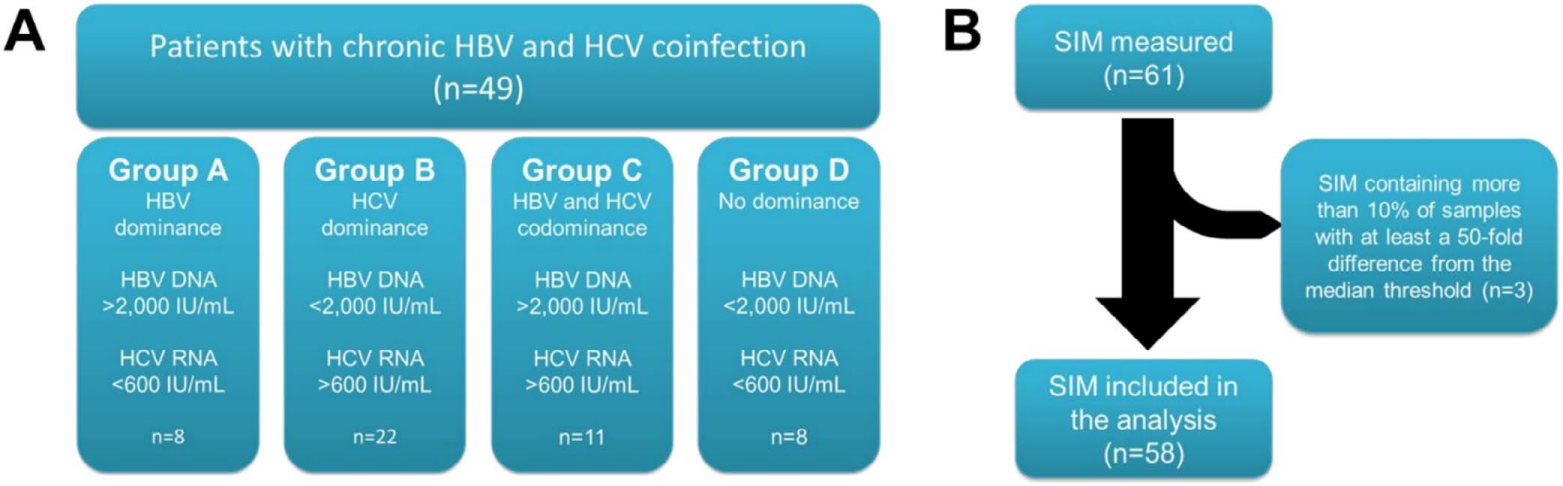
Classification of study cohort according to dominance pattern. Overview of the study cohort and exclusion criteria for the measured SIM. (A) Subgroups were analyzed based on established dominance patterns. (B) Quality assessment of all measured SIM led to removal of three SIM from our analysis.

Forty‐nine patients with HBV/HCV co‐infection were recruited at Hannover Medical School between 1998 and 2008 in this retrospective study. All patients tested positive for HBV surface antigen (HBsAg) and anti‐HCV. Patients that received antiviral treatment at the time of sampling were excluded from our analysis. Samples were drawn at the outpatient clinic at regular follow‐ups, and patients were not hospitalised at the time of sampling. We classified all patients based on serological patterns, previously described by Raimondo et al. [16]: Group (a) HBV dominance with HBV‐DNA > 2000 IU/mL and HCV‐RNA < 600 IU/mL; (b) HCV dominance with HCV‐RNA > 600 IU/mL and HBV‐DNA < 2000 IU/mL; (c) HBV and HCV codominance with HBV‐DNA > 2000 IU/mL and HCV‐RNA > 600 IU/mL; (d) no virus dominant with HBV‐DNA < 2000 IU/mL and HCV‐RNA < 600 IU/mL (Figure 1).

These patients are all part of a previously published study by Wiegand et al. [20].

### 2.2 Definition of Liver Cirrhosis

Diagnosis of cirrhosis was either based on liver histology or non‐invasive methods such as ultrasound, FibroScan, or biochemical results. Liver cirrhosis in biopsies was defined as F4 in Metavir or F5–6 in Ishak. Liver elastography ≥ 12.5 kPa was considered as cirrhosis [21].

Patients with at least two of the following criteria: platelets < 100/nL, aspartate aminotransferase/alanine aminotransferase (ALT) ratio > 1, bilirubin > 1.5 upper limit of normal range, and albumin < 35 g/L fulfilled the biochemical assessment of cirrhosis. Individuals were considered to have cirrhosis if one of the definitions above was satisfied.

### 2.3 Sample Storage and Preparation

After blood draw was performed at the outpatient clinic of Hannover Medical School between January 1996 and December 2010, all samples were processed according to standard operating procedures. EDTA whole blood was centrifuged and plasma aliquots stored at a minimum of −20°C.

The concentrations of 61 SIM were measured in serum and plasma using the Luminex‐based multiplex technology (Bio‐Rad, Hercules, USA). The assay was performed according to the manufacturer's protocol. In brief, the cytokine standard was resuspended and diluted to generate standard curves for each analyte according to the instructions. The bead mixture was incubated for 30 min at room temperature (RT) with 50 μL standard or serum samples that were diluted in sample diluent (1:2 dilution). Several washing steps were performed with 100 μL wash buffer per well, using the automated washer for magnetic beads. After the addition of the secondary biotinylated antibody mix for 30 min at RT and three more washing steps, Streptavidin‐Phycoerythrin was added for 10 min at RT (1:100 dilution). After three final washing steps, the beads were resuspended with 125 μL assay buffer and the data were acquired.

The Cobas Amplicor HCV Monitor Test (Roche, Basel, Switzerland) was used to detect HCV‐RNA. The quantitative cut‐off was 600 IU/mL. The qualitative cut‐off was 50 IU/mL.

### 2.4 Quality Control of Acquired Data

An assessment of outliers in the data set was performed before conducting further analysis. Therefore, a 50‐fold median threshold and a 1/50th median threshold were used in each group to detect outliers, as previously described [22]. Outliers were excluded from further analysis. Based on this assessment, three SIMs that contained more than 10% of samples considered as outliers were excluded. The remaining 58 SIMs were included in the final analysis (Figure 1).

In addition, to control a possible bias from different types of blood samples, a correction factor was calculated based on the median concentration of each SIM as described previously [22]. In detail, the quotient of median concentration of SIM in plasma divided by median concentration of SIM in serum results was built. Thereafter, each serum result was multiplied by the correction factor.

### 2.5 Statistical Analysis

To perform statistical analysis, R Statistical Software (Version 4.2) was used. All graphs were created with packages listed in Table S1. To analyse differences between independent groups, the Mann–Whitney *U* test was used. All *p* values were adjusted using the Benjamini–Hochberg procedure with a false discovery rate of 5%, and all correlations were calculated using the Pearson method. We performed a Kruskal–Wallis test to detect differences in the baseline characteristics between all groups.

In order to assess differences in the multidimensional data set, a principal component analysis (PCA) was performed using the prcomp function from the stats package. All samples that contained at least one SIM classified as an outlier (*n* = 16) were excluded for this analysis. For the PCA, we needed a complete data set on all SIM measured for each sample, and therefore, 33 samples were tested in our final analysis.

We used the STRING database in order to conduct our protein interaction analysis based on all altered SIM in HBV‐active patients (*n* = 22). Edges symbolize that SIM are part of the same physical complex. SIM that interacted on a high confidence level (> 0.7) were included in our analysis, and line thickness indicates the level of confidence of the interaction. The pathway analysis was conducted based on the same 22 SIM using the ConsensusPathDB database and included the top 15 most significantly (*p* < 0.05) altered Kyoto Encyclopedia of Genes and Genomes (KEGG) pathways.

We performed a sensitivity analysis using the sensemakr package in R to assess the impact of unobserved confounders. A linear regression model was created using the lm() function from the stats package. The complete model is presented in the supplements.

## 3 Results

### 3.1 Characteristics of the Study Cohort

The study cohort included 49 patients chronically infected with HBV and HCV (Table 1). Patients were middle‐aged (mean 42.1 years) and predominantly male (33 male, 16 female). However, patients showing a codominant infection pattern were more often women (73%, *p* = 0.003). The frequency of cirrhosis in our cohort was 14.6% (*n* = 6). Patients with a codominant infection pattern showed the highest cirrhosis frequencies (*n* = 3, 43%, *p* = 0.03). Aspartate aminotransferase (AST) and alanine amino transferase (ALT) were highest in HCV‐dominant patients and patients without a dominance pattern. HBsAg and HBV‐DNA were highest in the two subgroups of patients showing HBV activity (HBV dominance and HBV/HCV codominance). In contrast, HCV‐RNA was highest in patients showing HCV activity (HCV dominance and HBV/HCV codominance). All measured SIM values are presented in Table S2.

**TABLE 1:** Baseline characteristics of the study cohort. Mean values and standard deviations are shown.

	HBV dominance	HCV dominance	HBV/HCV codominance	No dominance	Reference	*p* value
*n*	8	22	11	8		
Age	43.5 ± 16.6	41.9 ± 10.2	43.1 ± 18.2	39.6 ± 21.1		0.998
Sex	1 f 7 m	3 f 19 m	8 f 3 m	4 f 4 m		0.003
Cirrhosis (+)	2	1	3	0		0.03
Platelets (Tsd/μL)	149 ± 58	178 ± 71	201 ± 87	166 ± 95	160–370	0.59
AST (U/L)	29.7 ± 19.7	65.1 ± 48.0	23.3 ± 9.1	74.7 ± 94.3	0–35 (m) 0–31 (f)	0.17
ALT (U/L)	28.6 ± 9.7	121.7 ± 124.1	43.8 ± 23.4	54.0 ± 50.9	0–45 (m) 0–34 (f)	0.06
Bilirubin (μmol/L)	19.0 ± 19.8	15.7 ± 10.7	14.3 ± 10.7	21.4 ± 13.2	2–21	0.51
Gamma‐glutamyltransferase (U/L)	31.0 ± 18.1	99.4 ± 111.1	64.3 ± 66.1	88.0 ± 68.7	35–52	0.10
Alkaline phosphatase (U/L)	92 ± 60	114 ± 65	206 ± 144	141 ± 60	40–129 (m) 35–104 (f)	0.07
HBsAg(IU/mL)	37,905 ± 46,860	2433 ± 3246	29,446 ± 68,048	7046 ± 7301	0	0.002
HBeAg (+), *n* (%)	4 (50%)	0 (0%)	7 (70%)	2 (29%)	—	< 0.001
HBV‐DNA (IU/mL)	4,511,863 ± 8,047,402	66 ± 270	37,128,000 ± 80,835,308	17 ± 41	0	< 0.001
HCV‐RNA (IU/mL)	0.75 ± 0.46	1,193,888 ± 2,077,059	922,909 ± 1,418,164	77 ± 212	0	< 0.001

### 3.2 SIM Expression Is Distinct Based on Dominance Patterns

**FIGURE 2:**
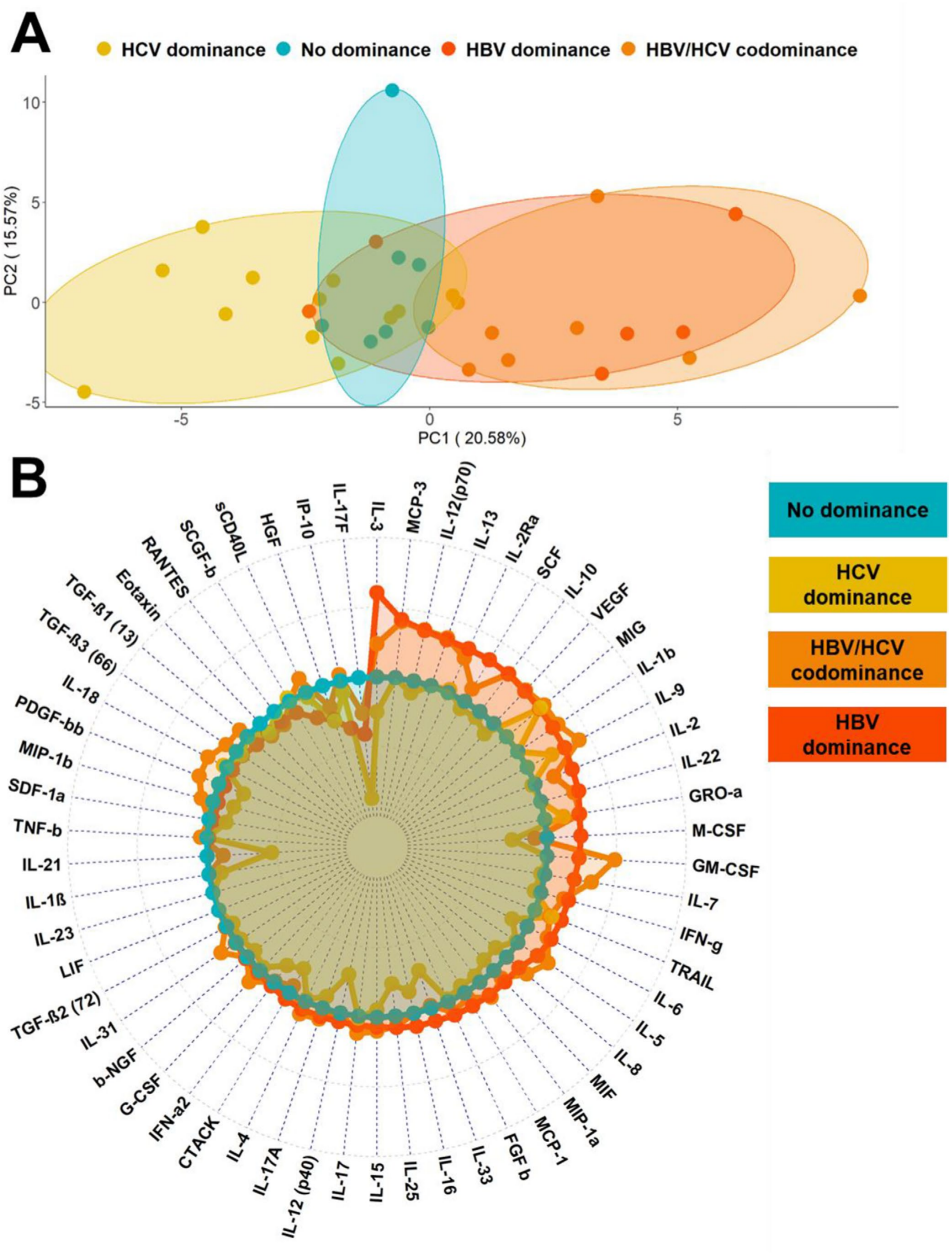
SIM expression was increased in HBV dominant groups and build a cluster. (A) All samples with outliers in the data set were removed to perform the principal component analysis (*n* = 33). Principal components 1 and 2 represent about 36% of the variance in the data set. (B) Estimated differences based on Mann–Whitney *U* test were used and HCV‐ (yellow), HBV‐ (red) and codominant (orange) patterns were compared with patients who showed no dominance pattern (turquoise).

In order to detect potential differences on a large scale between dominance patterns, a PCA of all SIM (*n* = 59) was performed. Principal components 1 and 2 represented together 36.2% of the variance in the data set. Patients with HBV dominance and HBV/HCV codominance exhibited similar SIM expression, forming a cohesive cluster. In contrast, those with an HCV dominance pattern clustered on the opposite side (Figure 2A).

We estimated the differences between dominance patterns for a detailed analysis based on the Mann–Whitney U test. Coinfected patients, who are classified as HBV dominant or HBV/HCV codominant, had higher concentrations of IL‐3 (*p* = 0.013), IL‐12(p70) (*p* = 3.82 × 10^−4^), IL‐13 (*p* = 0.019) and IL‐2RA (*p* = 0.013) compared to patients without a dominance pattern (Figure 2B).

### 3.3 SIM Expression Is Associated With HBV Activity

**FIGURE 3:**
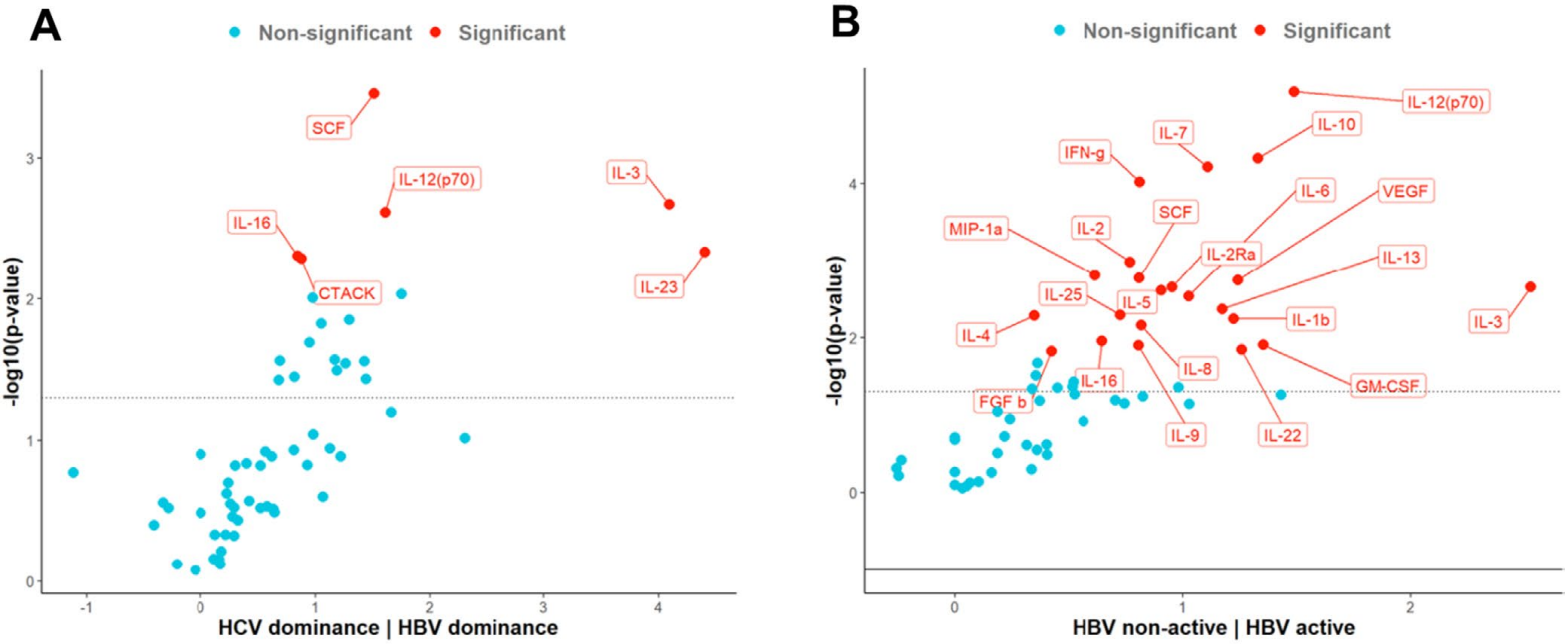
HBV dominance and activity is associated with overall SIM expression. (A) HBV dominance was compared to HCV dominance and (B) HBV activity (HBV dominance and HBV/HCV codominance patterns) was compared to patients without HBV activity (HCV dominance and no dominance patterns). Estimated differences (x‐axis) and adjusted *p* values (y‐axis) are shown and the dashed line represents the unadjusted significance level (*p* = 0.05).

To investigate the activity of dominance patterns, SIM concentrations for the main dominance pattern (HBV dominance vs. HCV dominance) were compared. Patients with an HBV dominance pattern showed significant SIM alterations in comparison to patients with an HCV dominance pattern. HBV dominant patients had higher expression of IL‐16, CCL27/CTACK, SCF, IL‐12(p70), IL‐3 and IL‐23 (adjusted *p* < 0.05). We measured the highest differences for IL‐3 and IL‐23 in comparison between both groups (Figure 3A).

Frequency of SIM alterations was highest between patients with HBV activity (HBV‐DNA > 2000 IU/mL; HBV dominance and HBV/HCV codominance) compared to patients with less HBV activity (HBV‐DNA < 2000 IU/mL; HCV dominance and no dominance). Overall, 22 SIM were increased in patients with HBV activity, including IL‐3, IL‐12(p70), GM‐CSF, VEGF and IL‐10. Patients without HBV activity did not show any increased SIM expression (Figure 3B).

**FIGURE 4:**
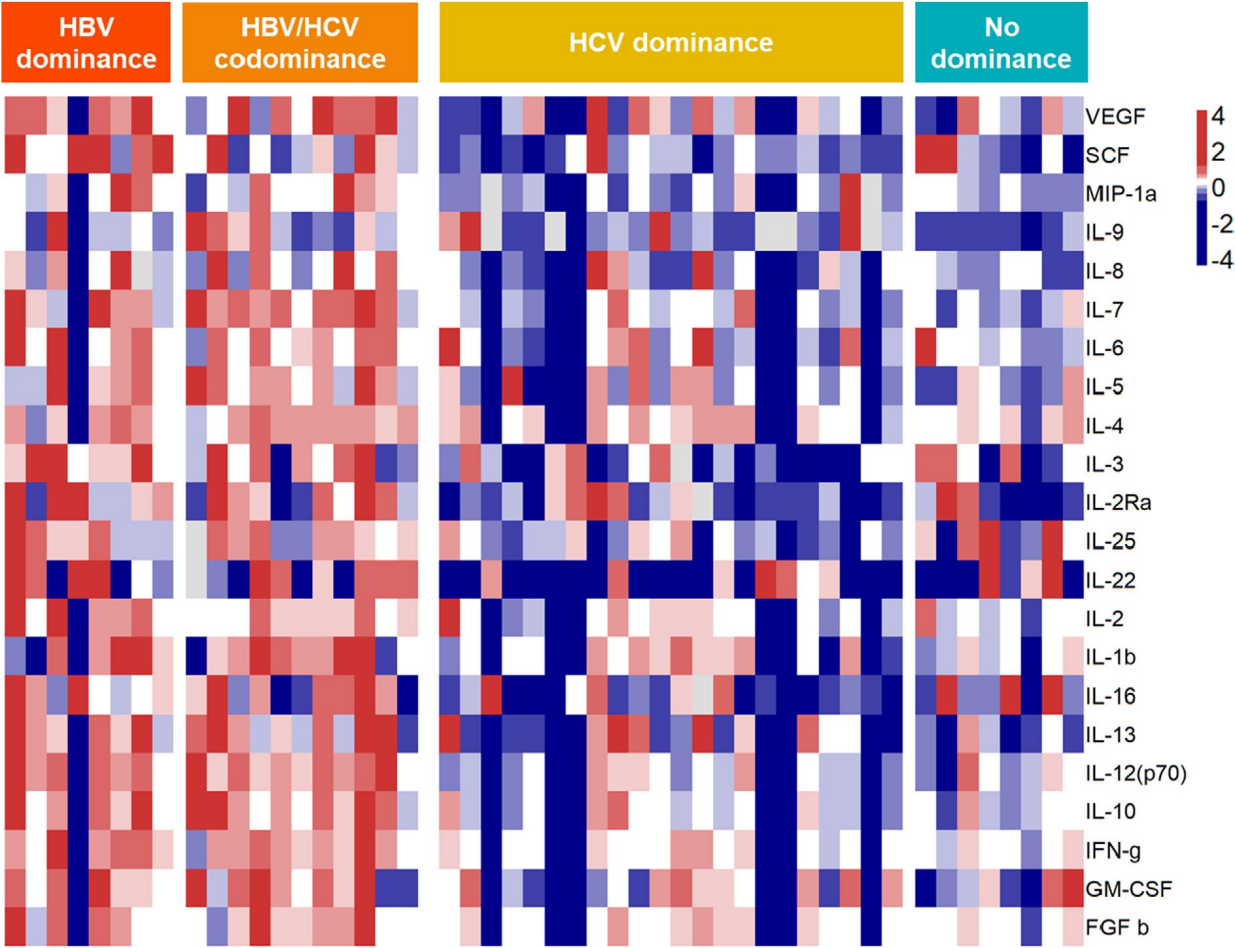
Significantly altered SIM led to cluster formation between dominance patterns in coinfected HBV/HCV patients. Quantile breaks show differences between high SIM expression (red) and low SIM expression (blue).

To better demonstrate the distribution of the significantly increased 22 SIM, a heat map analysis was performed (Figure 4).

### 3.4 SIM Altered due to HBV Activity Show High Interactivity

**FIGURE 5:**
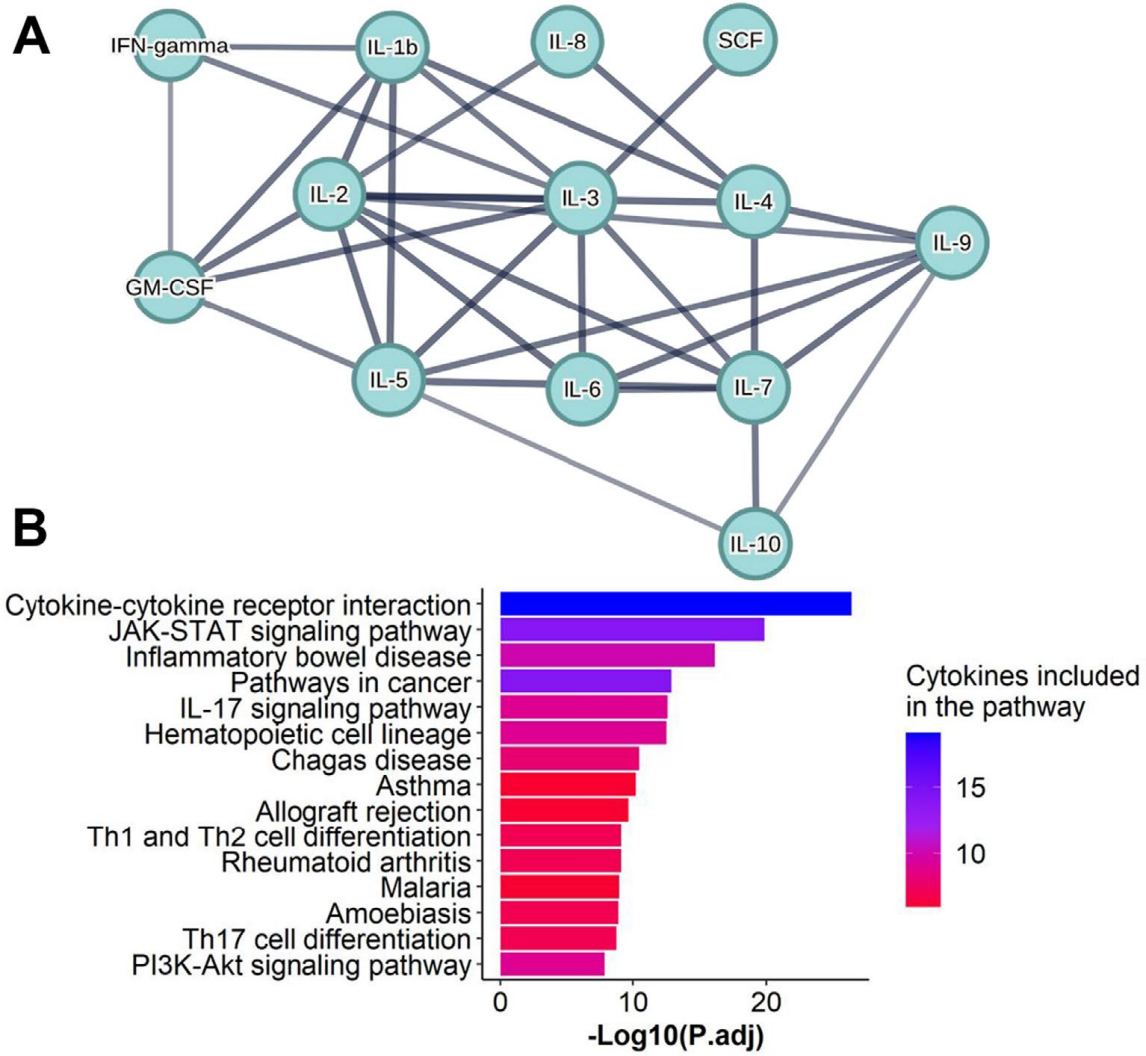
HBV activity was associated with SIM interactivity and induced various signal pathways. (A) Edges indicate that SIM are part of a physical complex and line thickness indicates the confidence level. (B) Pathway analysis of altered SIM (*n* = 22) based on HBV activity was performed by use of the KEGG database to calculate the top 15 altered pathways. The colours indicate how many altered cytokines are included in the pathway.

Interactivity analysis of altered SIM due to HBV activity led to a cluster of 13 out of 22 SIM based on a high confidence level (> 0.7). IL‐3 had the most interactions (*n* = 7), followed by interleukins IL‐5, IL‐2 and IL‐6. Interferon‐gamma, SCF and GM‐CSF were the only non‐interleukins involved (Figure 5A).

### 3.5 HBV Activity Is Associated With JAK–STAT and IL‐17 Signalling Pathways

To further investigate how HBV activity affects different pathways in HBV/HCV coinfection, a pathway analysis based on the Kyoto Encyclopedia of Genes and Genomes (KEGG) database was performed. Cytokine‐cytokine receptor interactions played a major role (*p* = 3.59 × 10^−27^) highlighting the high grade of interactivity. In addition, altered pathways included the JAK–STAT pathway, involved in immunity and cell death (*p* = 1.36 × 10^−20^) and pro‐inflammatory IL‐17 signalling (*p* = 2.47 × 10^−13^) as well as Th1/Th2 (*p* = 7.32 × 10^−10^) and Th17 (*p* = 1.69 × 10^−9^) cell differentiation (Figure 5B).

### 3.6 CCL27/CTACK and SDF‐1alpha Are Inversely Associated With HCV‐RNA

Correlation analysis of serological viral patterns with SIM was performed. Interestingly, CCL27/CTACK (*r* = −0.69, *p* = 7.02 × 10^−6^) and SDF‐1 alpha (*r* = −0.55, *p* = 0.002) correlated with HCV‐RNA negatively (Figure S1). No correlations between HBsAg or HBV‐DNA and SIM were found (Figures S2 and S3).

### 3.7 Cirrhosis Status Is Not Associated With SIM Levels of HBV‐Associated SIM

The overall prevalence of cirrhosis in our cohort was low (*n* = 6), but there was a statistically significant difference in the prevalence of cirrhosis between patients with and without HBV activity. It has been previously described that cirrhosis affects cytokine expression in HCV monoinfection before and after treatment with DAA [23]. Therefore, we performed an explorative analysis of whether cirrhosis status was associated with an increased SIM concentration in all SIM that were associated with HBV activity. There was a slight trend towards higher SIM concentrations in patients with cirrhosis. However, this trend was not significant in the end and not responsible for higher SIM concentrations in patients with HBV activity (Figure S4). There was no association between FIB‐4 score and SIM concentrations (Figure S5).

### 3.8 Interplay of Age, Sex and SIM Concentrations

There was a significant difference in sex (*p* = 0.003) but not age in our subgroups. It has been described previously that age can have an effect on SIM concentrations in the context of viral hepatitis [24]. Therefore, we performed a comprehensive analysis of the interconnection between age, sex and SIM concentrations. We observed 4/22 altered SIM that differed significantly based on sex (Figure S6). In contrast, we did not observe any significant associations between age and SIM levels (Figure S7).

### 3.9 Sensitivity Analysis of Unobserved Confounders

In order to assess the impact of unobserved confounders, we performed a sensitivity analysis on all significantly altered SIM. The median robustness value (RV_q = 1_) was 22.1%, meaning that unobserved confounders that explain more than 22.1% of the residual variance of both the treatment and the outcome are strong enough to bring the point estimate to 0. The median partial R^2^ (*R* ^2^_Y∼D|X_) was 5.9%, meaning that an extreme confounder explaining 100% of the residual variance of the SIM concentration would need to explain at least 5.9% of the residual variance of the dominance pattern to fully account for the observed estimated effect (Table S3).

## 4 Discussion

Our study results reveal that distinct virological dominance patterns are associated with a unique expression of soluble immune mediators in HBV/HCV coinfection. Notably, SIM expression is linked to HBV activity, shedding light on a previously underestimated role of HBV in patients with HCV coinfection.

Interestingly, PCA analysis demonstrated cluster formation of SIM from HBV‐dominant and codominant patients, suggesting that HBV may trigger SIM expression. HBV is commonly considered a stealth virus that does not induce an innate immune response in hepatocytes [25, 26]. However, conflicting data from in vitro and in vivo studies exist that also suggest that HBV is able to induce innate immune responses. For example, Zhang et al. showed that infection of primary human hepatocytes led to immune responses that were similar to those induced by toll‐like receptor 2 [27].

However, what argues in favour of HBV as a stealth virus is the finding that the HBV markers HBsAg and HBV‐DNA were not associated with the SIM level. This may indicate that the inflammatory milieu is not directly induced by HBV, but that the inflammatory milieu itself induces different types of dominance patterns. For example, it has been shown that single nucleotide polymorphisms in the Th17 cell‐related RAR‐related orphan receptor C (RORC) gene may be associated with spontaneous HCV clearance [28]. This could mean that genetic host factors may determine virological dominance patterns in coinfected patients.

Pathway analysis identified associations between HBV activity and key immune pathways, including the JAK–STAT signalling pathway and the Th17/IL‐17 axis. These pathways have been implicated in immune regulation and liver disease progression in patients chronically infected with HBV [29]. This is of great importance, since HBV and HCV coinfection are associated with even higher incidences of cirrhosis and HCC than monoinfection alone [4, 5]. The Th17/IL‐17 axis is known to promote liver fibrosis through IL‐6 and IL‐23 expression and activation of hepatic stellate cells [30]. In our cohort, cirrhosis was more frequent in patients with HBV dominance or codominance, though the sample size was limited. Interestingly, 5/6 patients with cirrhosis in our study showed an HBV dominance or codominance pattern.

The JAK–STAT pathway is a major regulator of immune activation and has been previously linked to antiviral responses and liver fibrosis. STAT3 expression is associated with the development of liver fibrosis [31]. Regulation of immune cell activation is not limited to any certain immune cell type but is associated with the activation of many different immune cells [32, 33]. Interestingly, previous studies showed that the JAK–STAT pathway interacts directly with HBV and HCV replication and that activation of the pathway led to reduced replication [34, 35]. It is reasonable to suggest that activation of the JAK–STAT pathway is also one of the mechanisms involved in the establishment of different dominance patterns.

Additionally, we observed an inverse correlation between HCV‐RNA and certain immune mediators, such as CCL27/CTACK and SDF‐1alpha. While CCL27/CTACK has been associated with T cell–mediated inflammation [36, 37], its role in viral infections remains unclear. Similar findings have been reported in chronic hepatitis D virus infection [38], suggesting that viral replication levels may influence its expression.

A potential limitation of this study is its retrospective cross‐sectional study design. Moreover, the number of available patients in low‐frequent serological patterns was limited, unevenly distributed, and differences in sex, cirrhosis status and age between the groups of patients existed. We performed a subgroup analysis and demonstrated that sex, cirrhosis status and age are not confounding our results substantially. However, the effects of sex, cirrhosis status and age on SIM concentrations cannot be ruled out due to the small cohort size, limiting our ability to analyse these subgroups comprehensively. Additionally, information about comedications was limited, which may also bias SIM profiles. Samples were collected in the time before DAA therapy became available. Therefore, changes in viral epidemiology and treatment standards might impact our results. Nevertheless, this study takes into account the established dominance patterns and presents first insights into the soluble inflammatory milieu in patients coinfected with HBV and HCV. Prospective, observational studies are difficult to justify regarding the overall efficacy of DAA. Patient recruitment is very challenging, as the overall prevalence of untreated patients in a high‐income country like Germany is very low. Unfortunately, we were not able to include healthy controls or monoinfected patients and perform genetic studies to evaluate whether SNPs are associated with viral dominance patterns. The absence of a healthy control cohort is a major limitation of the study design. However, others have shown before that SIM expression is higher in patients with chronic hepatitis C and occult HBV infection than in healthy subjects [39]. Future studies should aim to unravel if viral dominance is the cause or consequence.

In summary, HBV dominance was associated with soluble immune mediators, especially involved in the Th17/IL‐17 axis and the JAK–STAT pathway. Both pathways are linked to immune regulation and disease progression. Their impact on the natural course of coinfection remains the subject of current and future studies.

## Author Contributions

M.C. and S.B.W. conceived the project, coordinated the analyses and designed the experiments. C.O., H.W., C.S.F., A.R.M.K., S.B.W. and M.C. drafted the manuscript. M.C., K.D. and H.W. were involved in the recruitment of patients. S.B.W. was involved in the creation of the clinical cohort and acquired the data with the help of A.W. C.O. and M.W. analysed the data, supervised by M.C. and S.B.W. All authors read and approved the manuscript.

## Ethics Statement

This article does not contain any studies with animals performed by any of the authors. The study protocol conformed to the ethical guidelines of the Declaration of Helsinki, and the local ethics committee approved this study a priori (No. 1407‐2012). Informed consent was obtained from all individual participants included in the study.

## Conflicts of Interest

M.C. reports personal fees from Abbvie, personal fees from Falk Foundation, personal fees from Gilead, personal fees from GlaxoSmithKline, personal fees from Jansen‐Cilag, personal fees from Merck/MSD, personal fees from Novartis, personal fees from Roche, personal fees from Spring Bank Pharmaceuticals, and personal fees from Swedish Orphan Biovitrum, outside the submitted work. H.W. reports grants/research support and personal fees from Abbvie, Biotest AG, and Gilead. He received personal fees from Aligos Therapeutics, Altimmune, Astra Zeneca, Bristol‐Myers‐Squibb, BTG Pharmaceuticals, Dicerna Pharmaceuticals, Enanta Pharmaceuticals, Dr. Falk Pharma, Falk Foundation, Intercept Pharmaceuticals, Janssen, Merck KGaA, MSD Sharp & Dohme GmbH, MYR GmbH, Norgine, Novartis, Pfizer Pharma GmbH, Roche and Vir Biotechnology, outside the submitted work. K.D. reports research and travel support from Gilead, and lecture fees from Gilead, AbbVie, and Altimmune outside the submitted work. C.O., M.W., A.W., A.R.M.K., C.F. and S.B.W. declare no conflicts of interest.

## Supporting information


**Data S1:** Supporting Information.

## Data Availability

The data that support the findings of this study are available from the corresponding author upon reasonable request.
