# Supplementary material for: HBV Dominance Is Associated With a Distinct Inflammatory Milieu in HBV/HCV Coinfection
Source: J Viral Hepat. 2025 Oct 15;32(11):e70092. doi: 10.1111/jvh.70092 (PMC12522073; doi:10.1111/jvh.70092)

**HBV dominance is associated with a distinct inflammatory milieu in HBV/HCV coinfection**

Carlos Oltmanns^1,2,3^, Moana Witte^1,2,3^, Anika Wranke^1,4,5^, Katja Deterding^1^, Heiner Wedemeyer^1,4,6^, Christine S Falk^4,7^, Anke R M Kraft^1,2,3,4,6^, Steffen B Wiegand*^8^, Markus Cornberg*^1,2,3,4,6^

^1^ Department of Gastroenterology, Hepatology, Infectious Diseases and Endocrinology, Hannover Medical School (MHH), Hannover, Germany

^2^ Centre for Individualised Infection Medicine (CiiM), a joint venture between the Helmholtz Centre for Infection Research (HZI) and Hannover Medical School (MHH). Hannover, Germany

^3^ TWINCORE, a joint venture between the Helmholtz-Centre for Infection Research (HZI) and the Hannover Medical School (MHH). Hannover, Germany

^4^ German Centre for Infection Research (DZIF), partner site Hannover-Braunschweig, Germany

^5^ HepNet Study-House/German Liver Foundation, Hannover, Germany

^6^ Cluster of Excellence RESIST (EXC 2155), Hannover Medical School, Carl-Neuberg-Straße 1, 30625 Hannover, Germany

^7^ Institute of Transplant Immunology, Hannover Medical School, Hannover, Germany

^8^ Department of Anesthesiology and Intensive Care Medicine, Hannover Medical School, Hannover, Germany.

*contributed equally

# Supplementary material:

Supplementary Table 1: R Packages used for visualization and analysis.

| **R Packages** |
| --- |
| ggrepel |
| tidyverse |
| OlinkAnalyze |
| dplyr |
| ggplot2 |
| stringr |
| pheatmap |
| readxl |
| ggVennDiagram |
| ggvenn |
| RColorBrewer |
| ggsignif |
| ggpubr |
| writexl |
| fmsb |
| paletteer |
| cowplot |
| gridExtra |
| scales |
| gplots |
| msigdbr |
| rstatix |
| remotes |
| ggradar |
| clusterProfiler |
| devtools |
| palmerpenguins |
| ggforce |
| sensemakr |

Supplementary Table 2: Measured SIM concentrations (pg/ml) for analyzed dominance patterns. Mean values are shown.


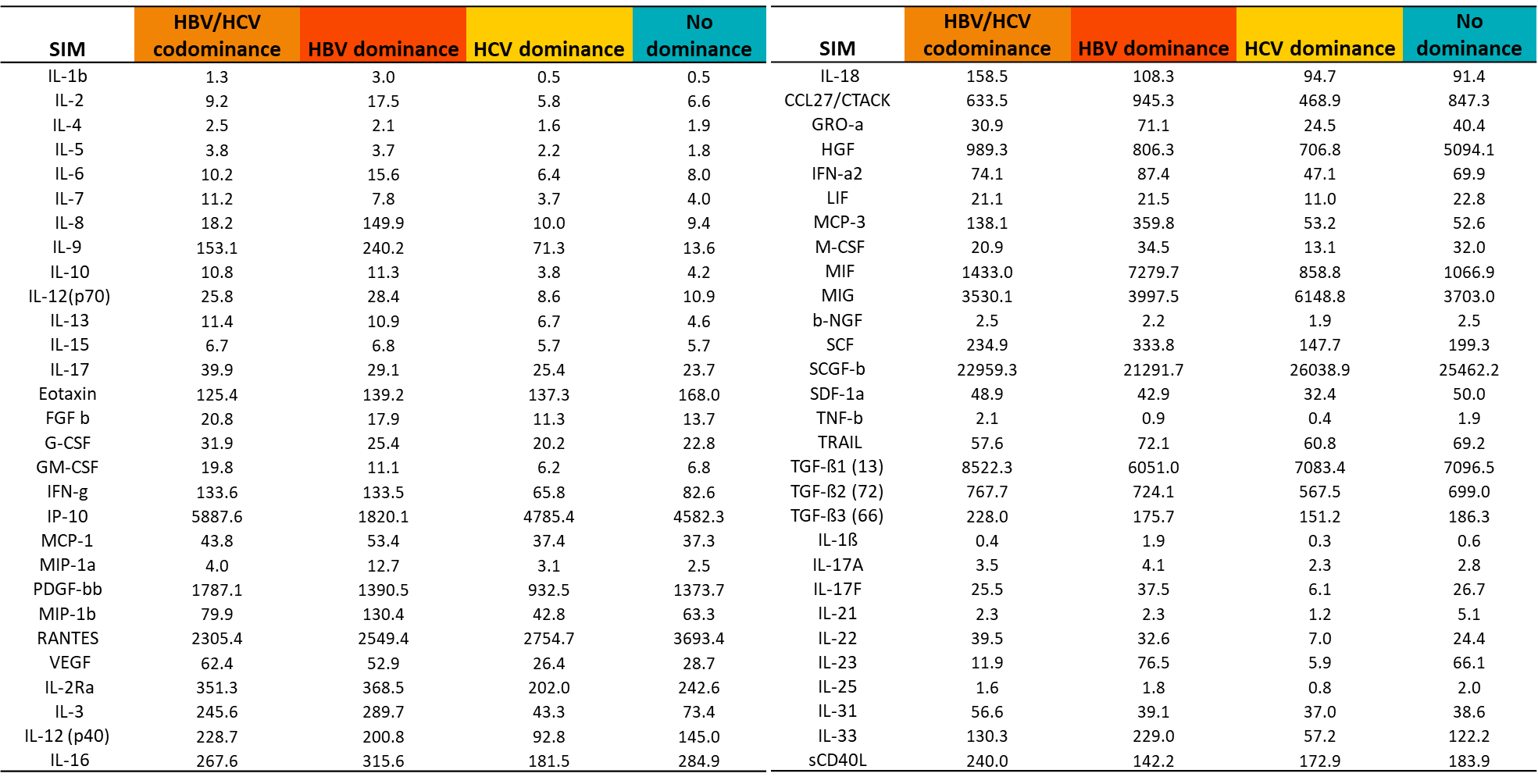


Supplementary Table 3: Sensitivity analysis to assess the potential impact of unaddressed confounders. Linear regression model was constructed using the formula shown below the table.

| SIM | Estimate | Standard error | t-value | *R^2^_Y∼D\|_*_X_ | *RV_q_*_=1_ |
| --- | --- | --- | --- | --- | --- |
| FGF-b | 0.81 | 0.86 | 0.94 | 5.9% | 22.1% |
| GM-CSF | 1.45 | 1.25 | 1.16 | 8.8% | 26.6% |
| IFN-g | 1.03 | 1.10 | 0.93 | 5.8% | 22.0% |
| IL-1b | 0.74 | 1.01 | 0.73 | 3.7% | 17.8% |
| IL-2 | 1.01 | 1.08 | 0.94 | 5.9% | 22.1% |
| IL-2Ra | 1.09 | 0.60 | 1.79 | 19.9% | 38.9% |
| IL-3 | 4.36 | 1.75 | 2.49 | 32.3% | 49.2% |
| IL-4 | 0.51 | 0.95 | 0.54 | 2.0% | 13.4% |
| IL-5 | 0.24 | 0.71 | 0.34 | 0.8% | 8.7% |
| IL-6 | 1.17 | 0.87 | 1.35 | 11.5% | 30.2% |
| IL-8 | 1.07 | 0.51 | 2.10 | 24.0% | 42.6% |
| IL-9 | 0.95 | 1.44 | 0.66 | 3.5% | 17.4% |
| IL-10 | 0.98 | 0.90 | 1.08 | 7.7% | 25.0% |
| IL-12(p70) | 0.94 | 0.96 | 0.97 | 6.3% | 22.8% |
| IL-13 | 0.42 | 0.56 | 0.76 | 3.9% | 18.3% |
| IL-16 | 0.45 | 0.65 | 0.69 | 3.5% | 17.4% |
| IL-22 | 0.66 | 1.25 | 0.53 | 2.0% | 13.2% |
| IL-25 | 0.04 | 0.87 | 0.05 | 0.0% | 1.2% |
| MIP-1a | 1.10 | 0.86 | 1.29 | 10.6% | 29.0% |
| SCF | 0.75 | 0.57 | 1.31 | 11.0% | 29.5% |
| VEGF | 0.30 | 0.68 | 0.43 | 1.3% | 11.0% |

Ŷ_i_ = *β*_0_ + *β*_1_(Dominance pattern) + *β*_2_(Age) + *β*_3_(Sex) + *β*_4_(Cirrhosis status) + *β*_5_(Platelets) + *β*_6_(AST) + *β*_7_(ALT) + *β*_8_(Bilirubin) + *β*_9_(Gamma-glutamyltransferase) + *β*_10_(Alkaline phosphatase) + *β*_11_(HBsAg) + *β*_12_(HBV-DNA) + *β*_13_(HCV-RNA) + ε_i_

Ŷ_i_ = SIM concentration ε_i_ = error term

Supplementary Figure 1: Associations of all measured SIM concentrations with HCV-RNA. Pearson coefficients are shown.


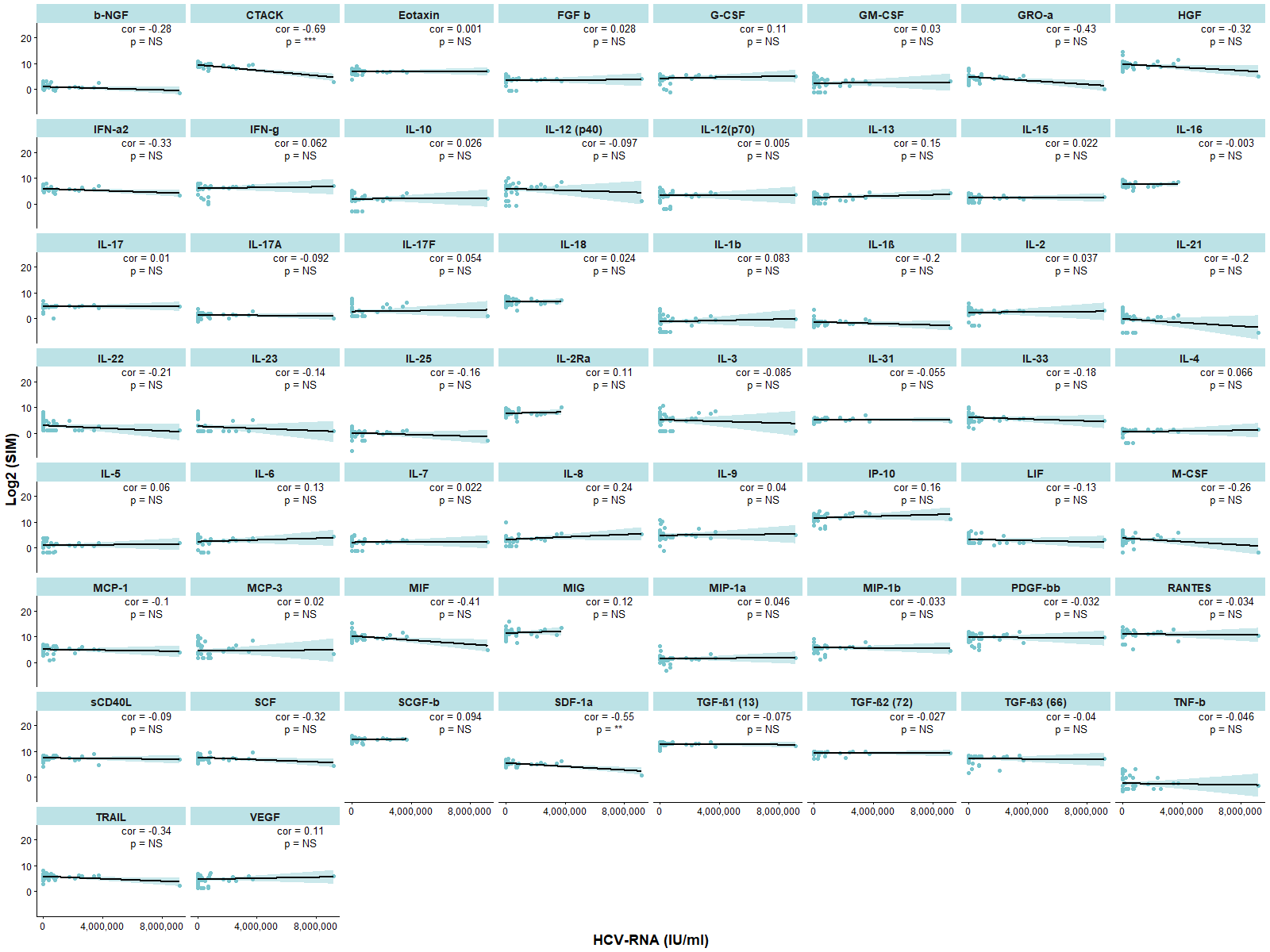


Supplementary Figure 2: Associations of all measured SIM concentrations with HBsAg. Pearson coefficients are shown.


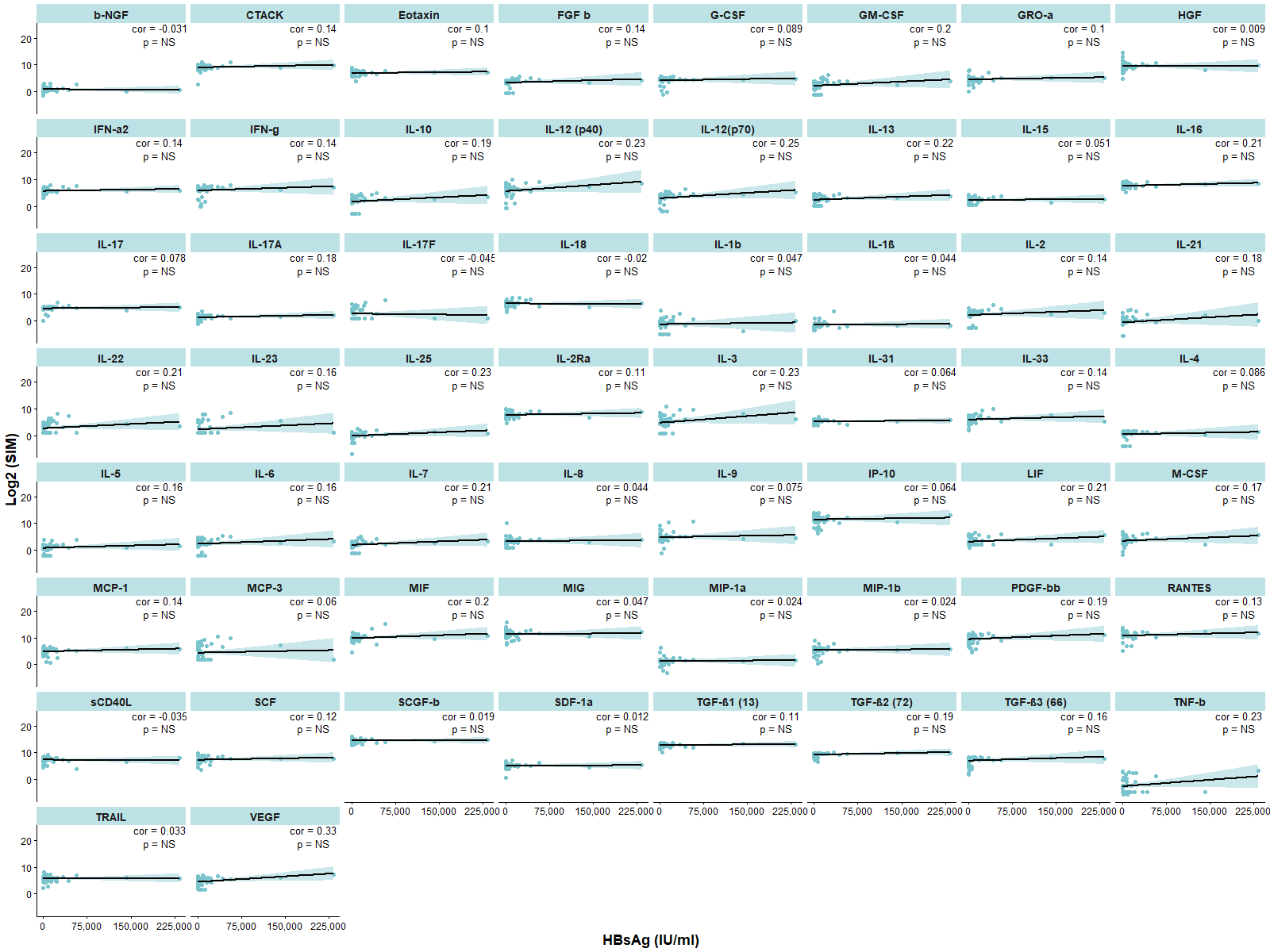


Supplementary Figure 3: Associations of all measured SIM concentrations with HBV-DNA. Pearson coefficients are shown.


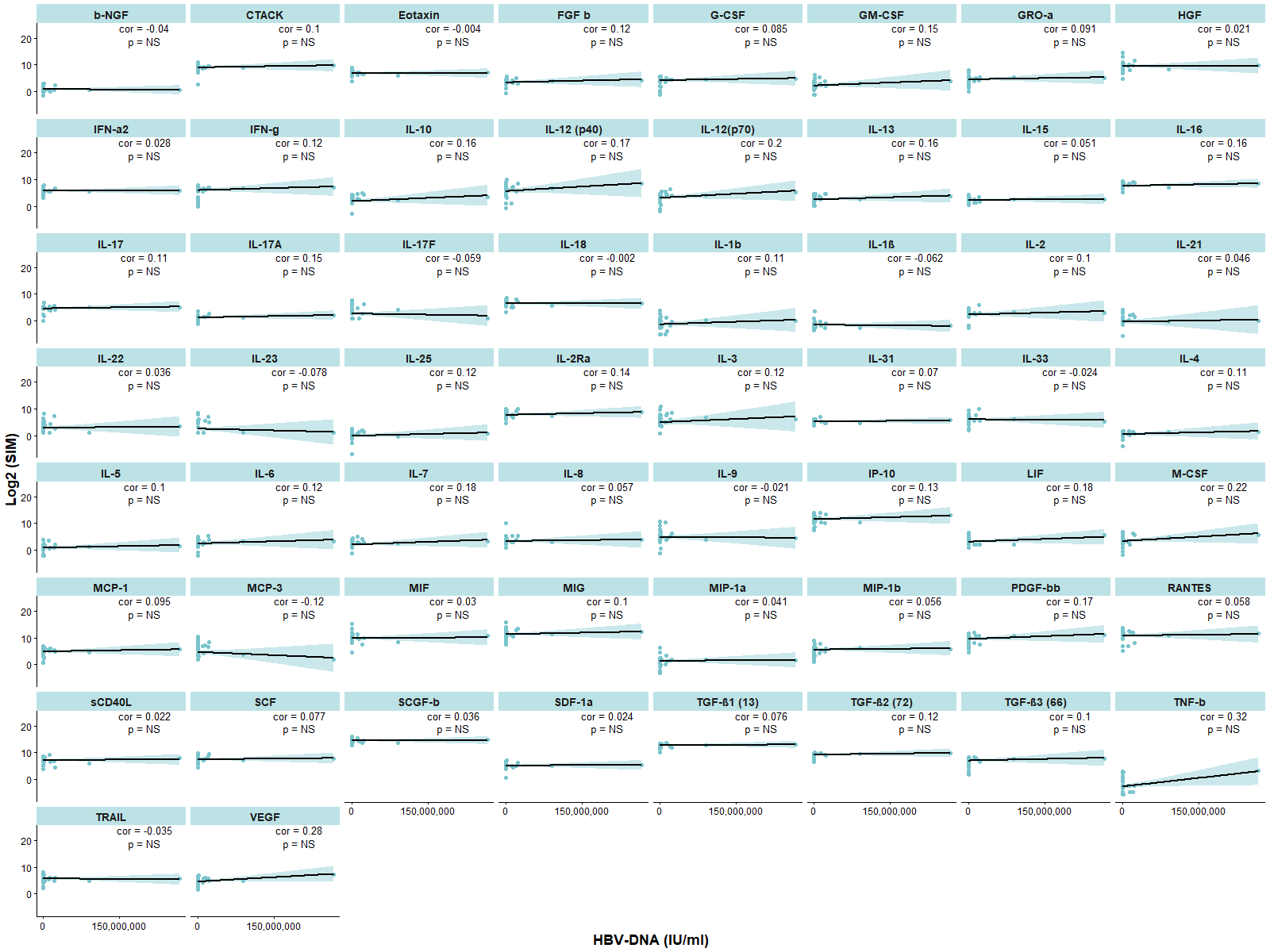


Supplementary Figure 4: Analysis of HBV-activity associated SIM based on cirrhosis status. Blue boxes and whiskers indicate patients without cirrhosis and red boxes and whiskers indicate patients with cirrhosis. Boxes indicate the interquartile distance and median, whereas whsikers indicate maximum and minimum values measured. SIM concentrations were tested using the Mann-Whitney-U test. P values were corrected for multiple testing using the Benjamini-Hochberg Procedure with a false discovery rate of 0.05%.There were no significant differences between patients with and without cirrhosis detectable.

***Supplementary Figure 5:*** Associations of FIB-4 score with significantly altered SIM concentrations between patients with and without HBV activity. Pearson coefficients are shown.


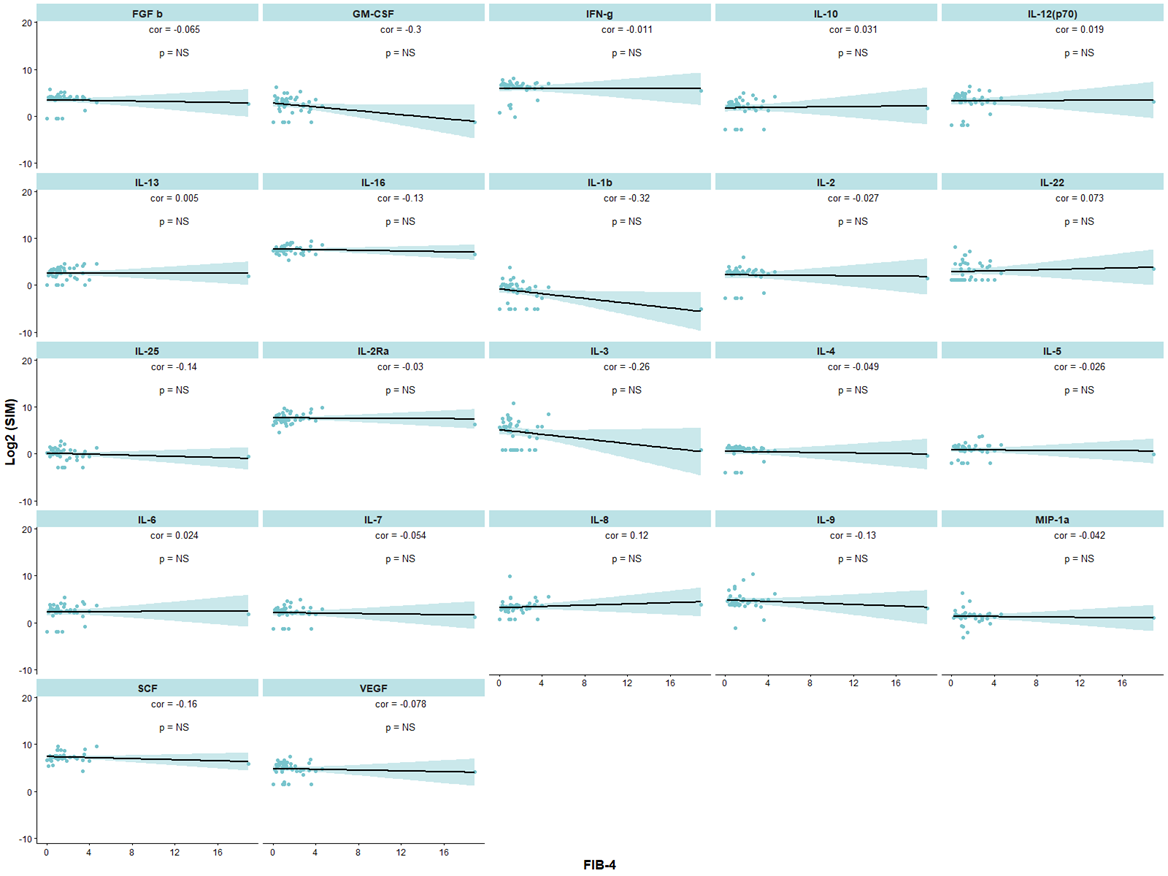


***Supplementary Figure 6:*** Analysis of HBV-activity associated SIM based on sex. Blue boxes and whiskers indicate male sex and red boxes and whiskers indicate female sex. Boxes indicate the interquartile distance and median, whereas whiskers indicate maximum and minimum values measured. SIM concentrations were tested using the Mann-Whitney-U test. P values were corrected for multiple testing using the Benjamini-Hochberg Procedure with a false discovery rate of 0.05%. ∗p <0.05.

***Supplementary Figure 7:*** Associations of age with significantly altered SIM concentrations between patients with and without HBV activity. Pearson coefficients are shown.


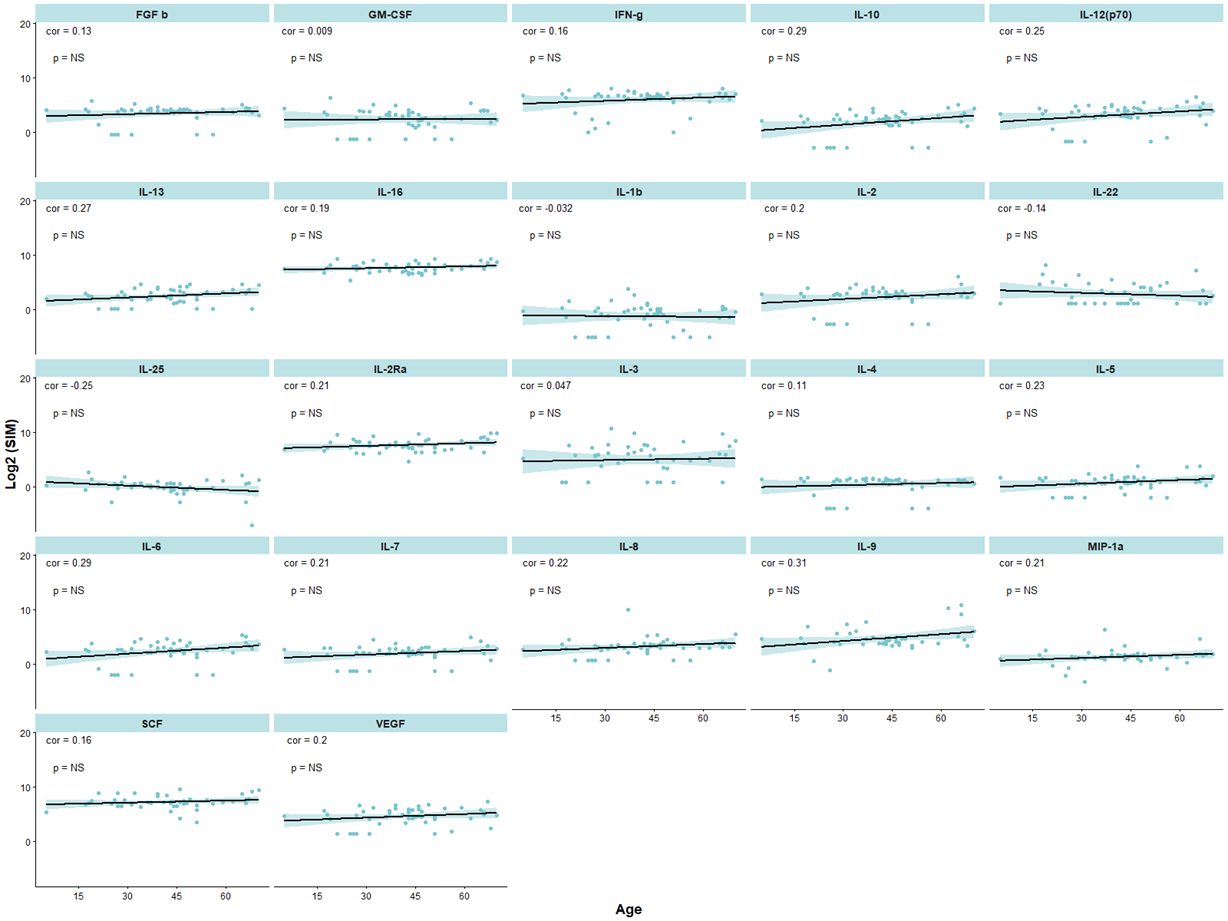

Supplement: Supplementary file 1 — Data S1: Supporting Information. [file JVH-32-0-s001.docx]
